# Intervention design involving persons living with dementia and care partners: Lessons learned from a design thinking workshop

**DOI:** 10.1002/alz70858_100537

**Published:** 2025-12-25

**Authors:** Cameron Gettel, James Galske, Chitra Dorai, Heidi Gil, Tonya Chera, Edith Stern, Kate Keefe, Erica DeFrancesco

**Affiliations:** ^1^ Yale School of Medicine, New Haven, CT, USA; ^2^ University of Connecticut School of Medicine, Farmington, CT, USA; ^3^ Amicus Brain Innovations, Inc., Chappaqua, NY, USA; ^4^ LiveWell Dementia Specialists, Plantsville, CT, USA

## Abstract

**Background:**

Persons living with dementia (PLWDs) and their care partners (CPs) have seldomly been included during early intervention development stages despite the potential for greater user engagement and adherence. Moreover, there is limited existing guidance for investigators to incorporate PLWDs and CPs in intervention design. In developing an emergency department care transition intervention, our goal was to conduct a Design Thinking Workshop (DTW), identify lessons learned, and provide recommendations for future intervention design processes targeting the population of PLWDs and CPs.

**Methods:**

Inclusive of PLWDs and their CPs, a total of 23 individuals participated in a DTW with components including empathy map review, preliminary technology intervention demonstration and feedback, current and future state discussion, among others. The design team collectively grouped challenges, successes, and processes of the DTW into 10 “lessons learned” with associated recommendations for effective inclusion of PLWDs and CPs.

**Results:**

The 10 lessons learned and associated recommendations were categorized into three groups, including pre‐workshop considerations (lessons 1 to 2), workshop principles and attitudes (lessons 3 to 7), and additive resources (lessons 8 to 10). Collectively, they are: (1) Recruit a participant pool resembling the target population; (2) Provide clear, detailed logistics information; (3) Ensure PLWDs and CP dyads are central in every aspect; (4) Dedicate sufficient time for introductions and contextualization; (5) Balance agenda items and time demands; (6) Select a lead facilitator(s) possessing direct experience with the target population and in design thinking; (7) Allow exercise flexibility; (8) Use multiple modalities to promote participant engagement; (9) Engage a transcriber and a visual artist; and (10) Ensure formal collection of post‐workshop feedback.

**Conclusions:**

Investigators including PLWDs and their CPs during intervention development phases may benefit from the Design Thinking Workshop lessons learned and recommendations to refine workshop practices and maximize end user engagement.

